# Effects of fatiguing, submaximal high‐ versus low‐torque isometric exercise on motor unit recruitment and firing behavior

**DOI:** 10.14814/phy2.13675

**Published:** 2018-04-19

**Authors:** Tyler W. D. Muddle, Ryan J. Colquhoun, Mitchel A. Magrini, Micheal J. Luera, Jason M. DeFreitas, Nathaniel D. M. Jenkins

**Affiliations:** ^1^ Applied Neuromuscular Physiology Laboratory Oklahoma State University Stillwater Oklahoma

**Keywords:** Firing Rate, Motor Unit Action Potential Amplitude, Muscle Fatigue, Recruitment Threshold, sEMG Decomposition

## Abstract

The purpose of this investigation was to evaluate the effects of repeated, high‐ (HT: 70% MVIC) versus low‐torque (LT: 30% MVIC) isometric exercise performed to failure on motor unit (MU) recruitment and firing behavior of the vastus lateralis. Eighteen resistance‐trained males (23.1 ± 3.8 years) completed familiarization, followed by separate experimental sessions in which they completed either HT or LT exercise to failure in random order. LT exercise resulted in a greater time to task failure and a more dramatic decline in the muscle's force capacity, but the total work completed was similar for HT and LT exercise. An examination of the firing trains from 4670 MUs recorded during exercise revealed that firing rates generally increased during HT and LT exercise, but were higher during HT than LT exercise. Furthermore, recruitment thresholds (RT) did not significantly change during HT exercise, whereas the RT of the smallest MUs increased and the RT for the moderate to large MUs decreased during LT exercise. Both HT and LT exercise resulted in the recruitment of additional higher threshold MUs in order to maintain torque production. However, throughout exercise, HT required the recruitment of larger MUs than did LT exercise. In a few cases, however, MUs were recruited by individuals during LT exercise that were similar in size and original (pre) RT to those detected during HT exercise. Thus, the ability to achieve full MU recruitment during LT exercise may be dependent on the subject. Consequently, our data emphasize the task and subject dependency of muscle fatigue.

## Introduction

Voluntary muscle force production is modulated by the systematic activation of motor units (MUs) of increasingly larger size in accordance with the size principle (i.e., MU recruitment) (Henneman 1957; De Luca and Erim 1994; Hu et al. 2013a) and by the alteration of the firing rates of already recruited MUs (i.e., rate coding). Larger MUs are activated at higher recruitment thresholds and portray greater action potential amplitudes (i.e., size) and twitch tensions in comparison to smaller, low‐threshold MUs (Milner‐Brown et al. 1973; Hu et al. 2013a). As a muscle fatigues over the course of sustained or repeated submaximal voluntary contractions, it has been suggested that active MUs increase their firing rates, and additional, higher threshold MUs are recruited (Adam and De Luca 2005; de Ruiter et al. 2005; Contessa et al. 2016; Mettler and Griffin 2016). These adaptations can be explained as the result of an increase in the excitation to the MU pool in order to maintain whole‐muscle force production in spite of fatigue‐induced decreases in MU twitch forces (Adam and De Luca 2003, 2005; de Ruiter et al. 2005; Contessa and De Luca 2013; Contessa et al. 2016).

Despite evidence for this understanding of MU behavior during fatigue (Adam and De Luca 2003, 2005; Contessa et al. 2016), previous studies have reported that during fatigue the firing rates of the majority of MUs decrease while new MUs are recruited (Enoka et al. 1989; Mottram et al. 2005; Vila‐Chã et al. 2012; Kelly et al. 2013; McManus et al. 2015). However, as noted by Contessa et al. (2016), it is possible that these conflicting results are due to the analysis of a small number of MUs that are recorded from intramuscular electromyographic (EMG) signals (Enoka et al. 1989; Mottram et al. 2005; Kelly et al. 2013) which are often grouped across contractions or force levels (Mottram et al. 2005; Vila‐Chã et al. 2012; McManus et al. 2015). It has been suggested that grouping MU data in this way misrepresents MU firing behavior (De Luca and Contessa 2012; Hu et al. 2013a). Furthermore, it is common practice to characterize MUs by their recruitment thresholds (RT) (Trevino et al. 2016; others), although fatigue most likely causes a reduction in MU RT. Consequently, fatigue studies that have characterized MUs in this way may mistakenly report a decrease in MU firing rate that is simply the consequence of observing a MU whose original RT was higher before fatigue.

Due to limitations in technology, it was previously very difficult or impossible to examine the behavior of large populations of MUs across a wide range of forces. Recently, however, noninvasive methods of MU recording have been developed, capable of extracting the activities of single MUs from the superficial surface of the skin. In this study, we utilize recently developed surface EMG (sEMG) recording and decomposition technology, originally described by De Luca et al. (2006) and improved upon by Nawab et al. (2010), to obtain the individual firings of dozens of MUs during fatiguing low‐ (30% maximal voluntary contraction strength (MVIC)) and high‐torque (70% MVIC) contractions. With the high accuracy and large number of detected MUs during isometric contractions, this technology has been proven advantageous in examining the behavior of MUs under varying conditions. For example, previous studies have characterized the behavior of the MU pool by examining the slopes and y‐intercepts of the MU firing rate versus RT relationship, MU firing rate versus MU amplitude relationship, and/or the MU amplitude versus RT relationship (Hu et al. 2013a; Pope et al. 2016; Trevino et al. 2016). Interestingly, these relationships have been shown to be sensitive to muscle fiber type composition (Trevino et al. 2016), training status (Herda et al. 2015) and resistance training (Pope et al. 2016), as well as force level (De Luca and Nawab 2011; Hu et al. 2013a, 2014a), and fatigue (Contessa et al. 2016).

Considering the aforementioned increase in excitation to the MU pool during fatiguing submaximal contractions, it has been hypothesized that resistance training with low‐loads to failure will result in activation of the entire MU pool (Mitchell et al. 2012; Potvin and Fuglevand 2017b). Recently, a model was developed by Potvin and Fuglevand (2017a) that predicted that all available MUs would be recruited during low‐force isometric contractions to fatigue. These investigators applied this model to compare MU behavior during simulated fatiguing low‐ (i.e., 20% MVIC) versus high‐force contractions (i.e., 80% MVIC), and predicted that low‐force contractions sustained to volitional fatigue would result in the eventual recruitment of the entire MU pool (Potvin and Fuglevand 2017b). However, it has been previously shown that, although muscle activation (i.e., EMG amplitude) increases throughout fatiguing submaximal isometric exercise, it may not reach maximal levels (Petrofsky et al. 1982; Fuglevand et al. 1993). Furthermore, the decrement in muscle activation at the end of submaximal, fatiguing exercise has been shown to be inversely proportional to the force‐level of contraction (Fuglevand et al. 1993; Jenkins et al. 2015). That is, muscle activation reaches higher levels during fatiguing high‐force (i.e., 80% MVIC) than low‐force contractions (i.e., 20% MVIC) (Petrofsky et al. 1982; Fuglevand et al. 1993; Alkner et al. 2000). Although EMG amplitude may not directly reflect neural drive to the muscle (Enoka and Duchateau 2015), these data may suggest a limitation in muscle excitation that is most dramatic during low‐force fatiguing submaximal contractions.

Given the conflicting reports of MU behavior during fatiguing exercise, the paucity of studies examining MU behavior during fatiguing high‐torque contractions, and the assertion that low‐torque contractions will cause recruitment of the entire MU pool (Potvin and Fuglevand 2017a), the purpose of this investigation was to evaluate the effects of repeated, high‐ (i.e., 70% MVIC) versus low‐torque (i.e., 30% MVIC) isometric knee extension exercise performed to failure on MU recruitment and firing behavior of the vastus lateralis. We hypothesized that: (1) fatigue would result in recruitment of larger, higher threshold MUs in both exercise conditions, (2) that MU firing rates would increase with fatigue, (3) that MU firing rates would be higher during the high‐ than low‐torque contractions, regardless of whether during early or late fatigue, and (4) that MUs with greater action potential amplitudes (MUAP_PP_) would be observed during high‐torque contractions, compared to the low‐torque condition.

## Methods

### Subjects

Eighteen resistance‐trained men completed this study, whose characteristics are described in Table 1. To be eligible for this study, each subject must have been: (1) between the ages of 18 and 39 years, (2) free from any physical limitations defined as any musculoskeletal injury, neuromuscular disorder, or chronic illness that may have limited exercise tolerance or performance, and (3) must have been resistance training their lower body for at least six consecutive months prior to the start of the study. Prior to participation, each participant signed an informed consent form and completed a health history questionnaire. This study was approved and carried out in accordance with the recommendations of the Oklahoma State University Institutional Review Board for the protection of human subjects (IRB Application #: ED‐16‐141).

**Table 1:** Subject Characteristics: Mean ± Standard deviation (SD)

	Age (yrs.)	Height (cm)	Weight (kg)	FM (kg)	FM (%)	FFM (kg)	FFM (%)	Resistance‐training History (yrs.)
Mean	23.1	176.4	85.5	13.6	15.3	72.0	85.0	7.4
SD	3.8	6.6	11.0	8.1	7.9	7.9	8.3	4.5

### Experimental design

A randomized, repeated measures, within‐group design was used for this investigation. Each subject visited the laboratory three times. Each visit was separated by 48–96 h and occurred at the same time of day (±2 h). During the first visit (Visit 0), subjects completed body composition analysis and familiarization, which included practicing maximal isometric contractions and tracking target torque trajectories during maximal and submaximal trapezoidal ramp tracings at the target torques to be used at Visits 1 and 2. Upon arrival to the laboratory for Visits 1 and 2, subjects completed a 5‐min dynamic warm‐up on a cycle ergometer, maximal and submaximal isometric contractions followed by 10 min of rest, and then unilateral, isometric leg extension exercise to volitional failure at a high‐ (HT; 70% MVIC) or low‐torque (LT; 30% MVIC). The torque used was randomized for Visits 1 and 2. Throughout Visits 1 and 2, sEMG signals were collected with a 5‐pin dEMG array sensor placed over the vastus lateralis (VL) to obtain the firing events of single MUs (described below). Subjects were asked to refrain from partaking in any vigorous physical activity for 24 h or any outside lower body exercise for 48 h, as well as abstain from consuming any ergogenic aids (e.g., caffeine) prior to Visits 1 and 2.

### Isometric testing

Subjects were seated on an isokinetic dynamometer (Biodex System 4; Biodex Medical Systems, Inc. Shirley, NY, USA) with straps securing the trunk and pelvis, and the lateral epicondyle of the femur aligned with the input axis of the dynamometer. All isometric testing and exercise was performed at a knee angle of 120° extension. Isometric knee extension torque (Nm) was measured through the lever arm of the isokinetic dynamometer, with the pad positioned 3–4 cm above the medial malleolus. The torque signal was displayed in real‐time on an external computer monitor for visual feedback to ensure accurate torque trajectory replication.

Once secured in the isokinetic dynamometer, participants performed two, 5 sec maximal MVICs, with approximately 60 sec of rest provided between contractions to avoid fatigue. The greatest torque achieved during a 1 sec epoch during the MVICs was recorded as the maximal torque output (Nm) for each subject and was used to calculate the target torque trajectories during subsequent isometric, trapezoidal contractions. Furthermore, maximal sEMG amplitude during this MVIC was quantified from a parallel‐bar, bipolar, sEMG sensor with an interelectrode distance of 10 mm (Delsys DE‐2.1, Delsys, Inc., Natick, MA, USA) placed on the VL in accordance with the SENIAM recommendations (Hermans et al. 1999). Following MVIC testing, participants performed a single maximal isometric trapezoidal contraction by tracking a 100% MVIC target torque trajectory displayed on a computer monitor. During this maximal tracing, trajectories increased linearly at a rate of 20% MVIC sec^−1^ to 100% MVIC, where it was held for 6 sec before decreasing linearly at a rate of 20% MVIC sec^−1^ until returning to baseline.

### Fatigue protocol

**Figure 1:**
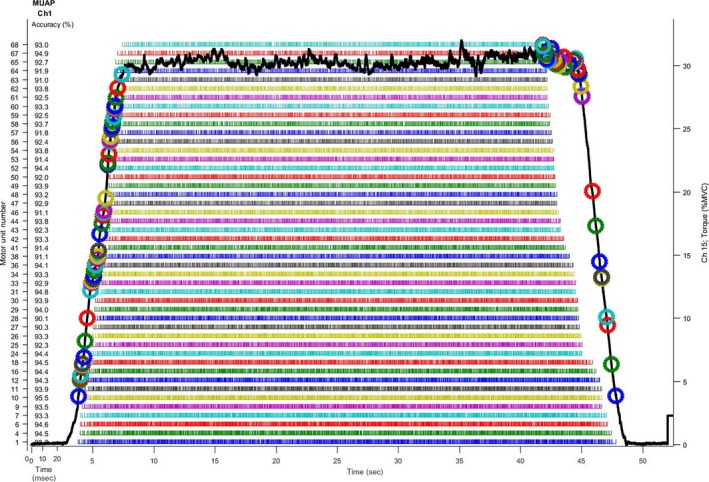
Example trapezoidal torque tracing (solid black line) and the accuracies, recruitment, and derecruitment thresholds (indicated by colored circles), and spike trains (rows of colored vertical lines under the torque tracing) of individual motor units for one subject from a repetition during the low‐torque exercise bout (i.e., 30% MVIC).

Following 10 min of rest, subjects then performed repeated submaximal, isometric trapezoidal contractions to failure by tracking target torque trajectories displayed on a computer monitor at either 70% MVIC (HT) or 30% MVIC (LT). During both conditions trajectories increased linearly at a rate of 10% MVIC sec^−1^ up to 70% or 30% MVIC, remained there for 7 sec or 37.2 sec, respectively, and then declined linearly at a rate of 10% MVIC sec^−1^ until returning to baseline. Subjects were instructed to maintain their torque output as close as possible to the displayed torque trajectory. An example force trajectory and force tracing at 30% MVIC are provided in Figure 1. Subjects performed these contractions until they could no longer consistently achieve the target torque level (as indicated by an inability to maintain torque within 5% of the target [HT: <65%, LT: <25%]). Approximately 6–7 sec of rest was provided between contractions during the HT and LT exercise bouts.

The number of repetitions performed during the HT and LT exercise were monitored and recorded. The total work performed during the HT and LT exercise bouts was calculated for each subject as the product of the number of repetitions performed, torque during contraction (Nm), and time (s) of contraction (note that the ramp up to and down from target torque were included in the calculation for work). Time to task failure was defined as the total time at the target torque during the HT (i.e., 70% MVIC) or LT (i.e., 30% MVIC) exercise for each subject.

### Motor unit recording and analysis

During the trapezoidal, maximal and submaximal (i.e. fatiguing) isometric contractions, sEMG signals were collected from the vastus lateralis using a five‐pin, four‐channel surface electrode array (Delsys, Inc., Natick, MA, USA). Prior to sensor placement, the surface of the skin was carefully prepared by shaving, removing superficial dead skin via abrasion and adhesive tape, and cleansing with alcohol. The sensor was secured to the skin with hypoallergenic tape at approximately two‐thirds of the distance between the center of the muscle belly toward the distal tendon (Zaheer et al. 2012) and a reference electrode was placed on the spinous process of the C7 vertebrae. The signals from the four channels of the dEMG array sensor were differentially amplified, filtered between 20 and 450 Hz, and sampled at 20 kHz using a 16‐channel Bagnoli EMG acquisition system (Contessa et al. 2016) (Delsys, Inc., Natick, MA, USA) and recorded on a computer for off‐line analysis.

Action potentials were extracted into the firing events of single MUs from the four separate EMG signals using the Precision Decomposition III (PDIII) algorithm described by De Luca et al. (2006) and improved upon by Nawab et al. (2010). The PDIII algorithm has been shown to reliability discriminate the discharge characteristics of large numbers of individual MUs during voluntary contractions up to maximal force levels (Nawab et al. 2010). Furthermore, the accuracy and validity of the decomposition methods used herein have previously been confirmed using both two‐source (i.e., intramuscular vs. sEMG) and reconstruct‐and‐test procedures (De Luca and Contessa 2012; De Luca et al. 2006; De Luca and Hostage 2010), and has been independently validated by Hu et al. (2013b,c, 2014b) using simulated signals and signals occurring in vivo.

The accuracies of the extracted firing instances for the detected MUs were tested using the Decompose‐Synthesize‐Decompose‐Compare test (De Luca and Contessa 2012), with only the recorded firing trains that achieved an accuracy ≥90% used for further analysis. The firing rate curves of each MU were computed by low‐pass filtering the impulse train with a Hanning window of 2 sec duration. Custom‐written software programs (Labview 2016; National Instruments, Austin, TX, USA) were used to calculate the following parameters for each validated MU:

1. *Recruitment threshold (RT)*: defined as the relative torque (%MVIC) at which the MU first discharged;
2. *Mean firing rate (MFR)*: calculated as the average firing rate (pulses sec^−1^ (pps)) during the plateau in each individual MU's firing curve; and
3. *Motor unit action potential amplitude (MUAP*_*PP*_ *)*: defined as the average peak‐to‐peak amplitude (mV) of the unique action potential waveforms from the 4 sEMG channels provided by the decomposition algorithm.

Both the shape and size of the MUAP waveforms provided by the PDIII algorithm agree with those derived using spike‐triggered averaging (Hu et al. 2013b). Furthermore, the size of the MUAP waveform (MUAP_PP_) has been shown to increase systematically with recruitment threshold in accordance with the size principle (Hu et al. 2013a, 2014a; Pope et al. 2016).

### Motor unit behavior during fatigue

To examine MU behavior during the fatiguing HT and LT exercise bouts, we examined MFR versus MUAP_PP_ and RT versus MUAP_PP_ relationships for each individual subject. In addition, MUAP_PP_ data from each condition (HT vs. LT) were normalized to maximal sEMG amplitude (calculated as the highest 500 ms EMG value obtained) during the prefatigue MVIC and were pooled and binned in 10% increments based on RT (%MVIC). This allowed us to further examine the changes in MUAP_PP_ within given RT ranges, across subjects, for HT versus LT exercise.

### Examination of maximal detected motor unit action potential amplitude during exercise

During both the high‐ and low‐torque fatigue protocols, the MU with the largest MUAP_PP_ was identified during the first, early, middle, late, and last repetitions of each condition, occurring at 0%, ~25%, ~50%, ~75%, and 100% of each subject's individual total number of repetitions, respectively, and utilized in subsequent analyses. First, we recorded its MUAP_PP_ and its RT. Second, we selected the MU with the greatest MUAP_PP_ for each subject during HT and LT exercise independent of repetition (maxMUAP_PP_) and predicted its original RT from an unfatigued, maximal contraction (as described below). Finally, we expressed the maxMUAP_PP_ relative to the subject's maximal predicted MUAP_PP_ from an unfatigued, maximal contraction (as described below).

### Predicted recruitment threshold and maximal motor unit size in unfatigued muscle

Each participant completed a maximal isometric ramp contraction using a trapezoidal trajectory prior to both the submaximal high‐ and low‐torque fatigue protocols in order to determine individual MUAP_PP_ versus RT and RT versus MUAP_PP_ relationships in unfatigued muscle. We used regression analyses to fit models to the MUAP_PP_ versus RT and RT versus MUAP_PP_ relationships. For most subjects, these relationships were fit with simple linear regression equations as follows:

(1) Y=b(X)+a

where *Y* was the predicted MUAP_PP_ or RT values, *X* was the RT or MUAP_PP_ values, b was the slope, and a was the y‐intercept. However, for several subjects, the relationships were better fit with a polynomial model and led to more accurate predictions. For these subjects, these relationships were represented as follows:

(2) $$Y=a\left(X^{2}\right)+b\left(X\right)+c$$

where *Y* was the predicted MUAP_PP_ or RT values, *X* was the RT or MUAP_PP_ values, *a* was the quadratic coefficient, *b* was the linear coefficient, and *c* was the constant. Therefore, these equations were determined for each individual subject from a prefatigue, maximal trapezoidal tracing on both the high‐ and low‐torque experimental testing days to account for potential interday variability in the relationships.In order to predict the original recruitment threshold of the maxMUAP_PP_ during the high‐ and low‐torque exercise, the maxMUAP_PP_ was entered as the X‐value in Equations 1 or 2 for the RT versus MUAP_PP_ relationships for each participant. This provided a Y‐value which was the predicted RT for a MU of comparable size during the un‐fatigued maximal contraction prior to exercise.

In order to determine how the maxMUAP_PP_ during the high‐ and low‐torque exercise compared to each participants’ maximal MUAP_PP_, an RT of 100 was entered as the X‐value in Equations 1 or 2 for the MUAP_PP_ versus RT relationships for each participant during each visit. This provided a Y‐value which was the largest (maximal) predicted MUAP_PP_ during the unfatigued maximal contraction prior to exercise. The maxMUAP_PP_ was then expressed as a percentage of the largest (maximal) predicted MUAP_PP_ (%Max_PRED_) for each subject during the high‐ and low‐torque exercise.

### Statistical analyses

Dependent samples t‐tests were used to compare MVIC strength and the largest predicted MUAP_PP_ between testing days, as well as the total repetitions, work performed, and time to task failure during the high‐ versus low‐torque fatiguing exercise bouts. The relationships between MVIC strength and time to task failure during the HT and LT conditions were analyzed with Pearson Correlation Coefficients using a one‐tailed test, because we hypothesized that time to task failure would increase as MVIC strength decreased. We also examined the relationships between the predicted original RT of the maxMUAP_PP_ during fatigue and time to task failure during the high‐ and low‐torque conditions with Pearson Correlation Coefficients. However, these relationships were examined using a two‐tailed test.

Linear regression analyses were performed to calculate slope and y‐intercept values for the MUAP_PP_ versus MFR and the MUAP_PP_ versus RT relationships for each individual subject and contraction during the HT and LT exercise (i.e., First, Early, Middle, Late, Last). Two separate two‐way repeated measures analyses of variance (ANOVAs) [Torque (70% MVIC vs. 30% MVIC) × Repetition (First vs. Early vs. Middle vs. Late vs. Last)] were used to examine differences among the slope and y‐intercept values. When appropriate, follow‐up analyses included one‐way ANOVAs and paired‐samples t‐tests. In addition, the slopes and y‐intercepts of these relationships at each repetition (First, Early, Middle, Late, Last) were regressed against the corresponding repetition during HT and LT exercise using polynomial regression to examine the pattern of change in the slopes and y‐intercepts across time in each condition. Finally, the mean slopes and intercepts of the individual relationships were used to construct the average linear regression line at each repetition during HT and LT exercise to more clearly illustrate the pattern of change across time during each condition.

The maximal detected MUAP_PP_ and its associated RT during the first, early, middle, late, and last repetitions during HT versus LT were analyzed with two separate two‐way repeated measures ANOVAs [Torque (70% MVIC vs. 30% MVIC) × Repetition (First vs. Early vs. Middle vs. Late vs. Last)]. When appropriate, follow‐up analyses included one‐way ANOVAs and paired‐samples t‐tests. The maxMUAP_PP_ and the predicted original RT of maxMUAP_PP_ during HT versus LT exercise were compared with dependent samples t‐tests. Likewise, the maxMUAP_PP_ expressed relative to the largest predicted MUAP_PP_ during HT versus LT exercise was compared with a dependent samples t‐test. For all analyses, an alpha of 0.05 was considered statistically significant.

## Results

### MVIC strength, max predicted MU size, repetitions, work, and time to task failure

**Figure 2:**
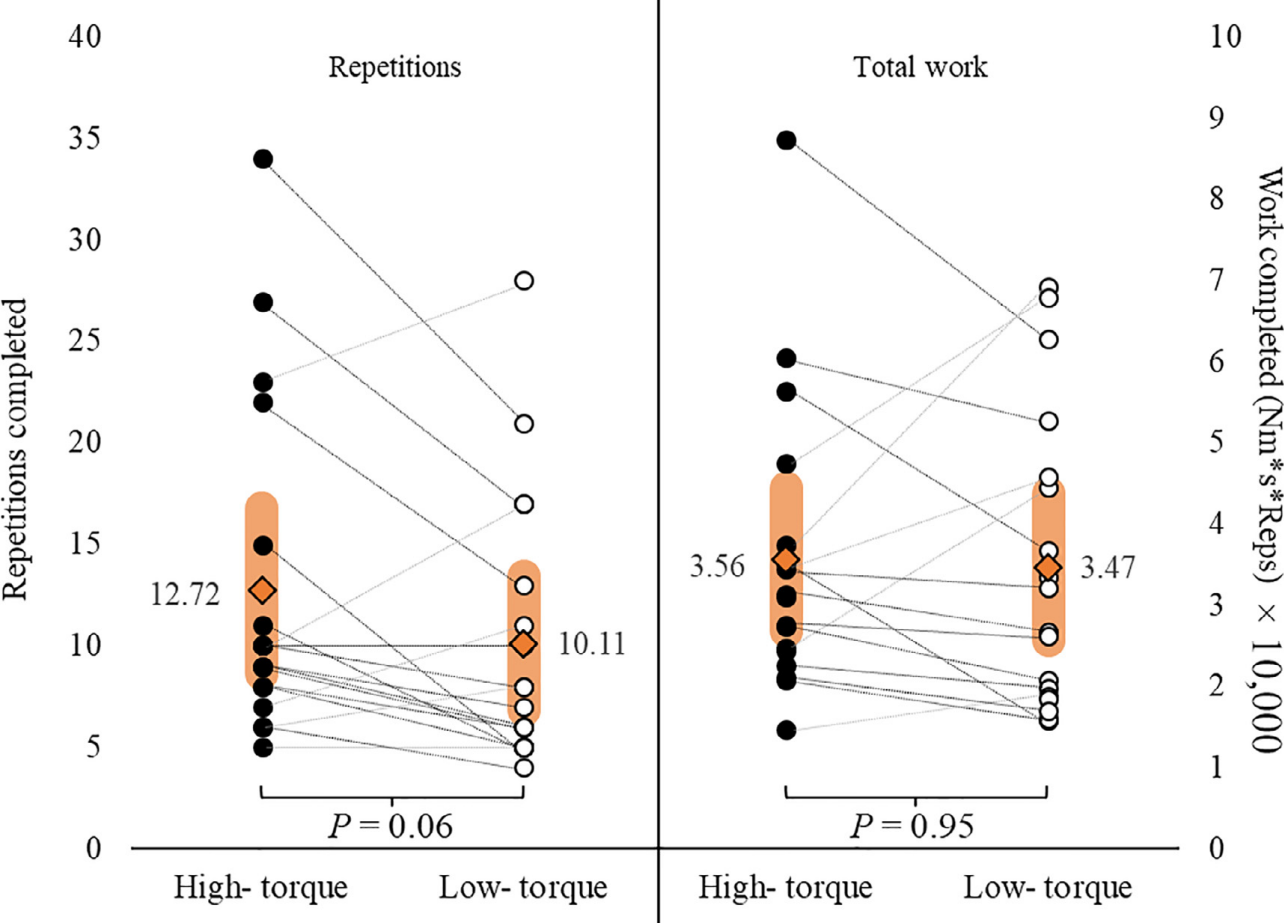
The individual responses and mean (orange filled diamonds) ± 95% confidence intervals (orange rounded bars) for total repetitions and total work completed during the high‐torque vs. low‐torque fatiguing work bouts.

**Figure 3:**
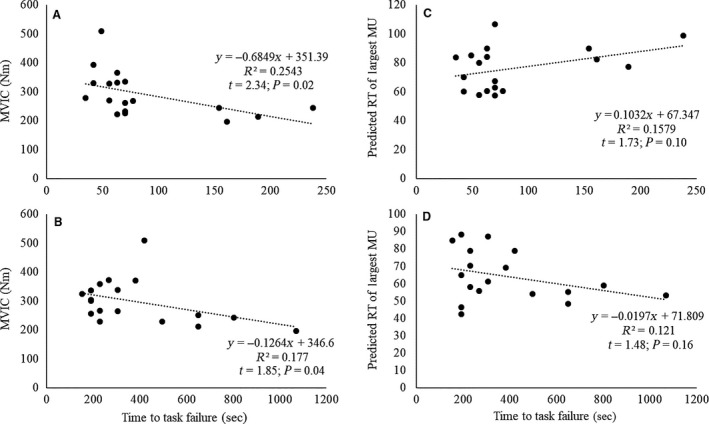
(A–B) Relationships between maximal voluntary isometric contraction (MVIC) strength and time to task failure during the (A) high‐torque and (B) low‐torque exercise. (C–D) Relationships between the predicted original recruitment threshold (RT; %MVIC) of the largest detected MU (maxMUAP_PP_) and time to task failure during the (C) high‐torque and (D) low‐torque exercise.

There was no difference in MVIC strength prior to HT versus LT exercise (mean ± 95% confidence interval = 291.72 ± 38.86 Nm vs. 297.76 ± 38.05 Nm, *t*_17_ = −1.11, *P *=* *0.28). Likewise, there was no difference in the largest predicted MUAP_PP_ between the HT versus LT testing days (753.26 ± 145.22 *μ*V vs. 777.06 ± 170.92 *μ*V; *t*_17_ = 0.29, *P *=* *0.77). The participants completed the same number of repetitions (12.72 ± 4.09 vs. 10.11 ± 3.31, respectively; *t*_17_ = 2.04; *P *=* *0.057) and the same amount of total work (35,565.31 ± 8809.42 au vs. 34,711.84 ± 9124.90 au, respectively; *t*_17_ = 0.24; *P *=* *0.95) during the HT versus LT exercise (Fig. 2). However, they had a greater time to task failure (386.24 ± 126.49 sec vs. 87.11 ± 28.61 sec, respectively; *t*_17_ = −5.97; *P *<* *0.001) during the LT versus HT exercise. MVIC strength was inversely related to time to task failure (Fig. 3) during both HT (*r* = −0.53; *P *=* *0.02) and LT (*r* = −0.42; *P *=* *0.04). The predicted original RT of the maxMUAP_PP_ was not significantly related to time to task failure during HT (*r* = 0.38; *P *=* *0.12) or LT (*r* = 0.35; *P *=* *0.16) exercise (Fig. 3C and D).

### Motor unit firing rate modulation during fatigue

**Figure 4:**
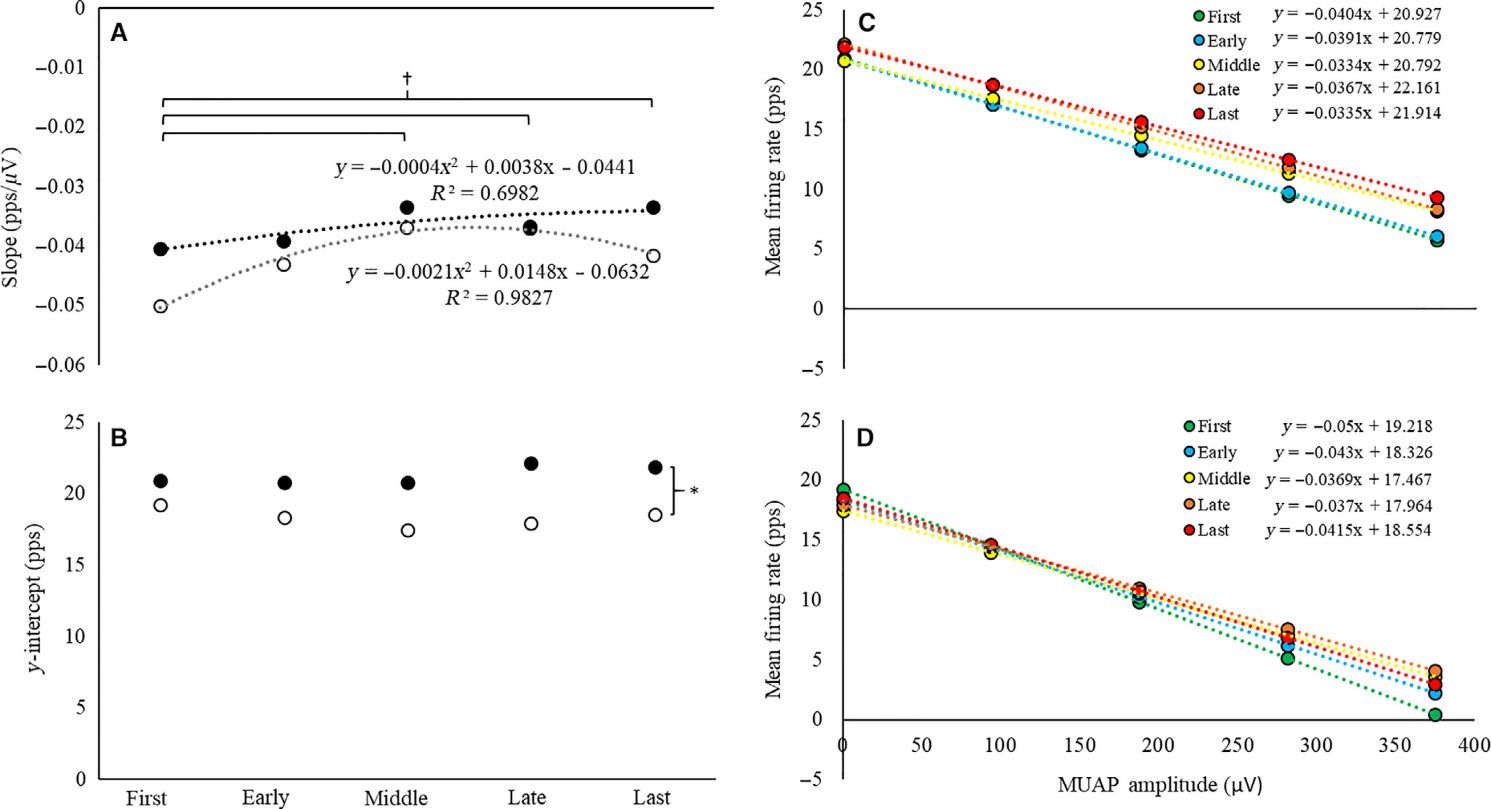
(A–B) The (A) slopes (pps *μ*V^−1^) and (B) y‐intercepts (pps) of the mean firing rate (MFR) versus motor unit action potential amplitude (MUAP_PP_) relationships during the first, early, middle, late, and last repetitions of the high‐torque (solid circles, black dotted line) and low‐torque (open circles, gray dotted line) exercise conditions. (C–D) The average linear regression lines for the MFR versus MUAP_PP_ relationship for the first, early, middle, late, and last repetitions during the (C) high‐torque and (D) low‐torque exercise. *indicates a significant main effect for torque (HT > LT); ^†^indicates a significant increase from the first repetition, independent of torque

Examination of the pattern of change for the average MFR versus MUAP_PP_ slopes (Table 2) indicated a slight, linear increase (*r* ^2^ = 0.70) in slope during HT and a quadratic relationship (*R* ^2^ = 0.98) characterized by an increase from the first to middle repetition, a plateau from the middle to late repetition, followed by a decrease from the late to last repetition during LT exercise (Fig. 4A). The ANOVA analysis indicated that there was no torque × repetition interaction (*F*_4,68_ = 1.13; *P *=* *0.35; $n_{p}^{2}$ = 0.06) or main effect for torque (*F*_1,17_ = 3.27; *P *=* *0.09; $n_{p}^{2}$ = 0.16), but there was a main effect for repetition (*F*_4,68_ = 4.55; *P *=* *0.003; $n_{p}^{2}$ = 0.21) for the average slopes of the individual MFR versus MUAP_PP_ relationships (Fig. 4A). The slopes increased (i.e., became less negative) from the first to the middle repetition (*P *=* *0.01), and then did not change from the middle to the last repetition (all *P *≥* *0.31).

**Table 2:** The individual slopes (pps *μ*V^−1^) and *y*‐intercepts (pps) of the mean firing rate (MFR) versus motor unit action potential amplitude (MUAP_PP_) relationships during the first, early, middle, late, and last repetitions of the high‐torque (HT) and low‐torque (LT) exercise conditions

	LT (30% MVIC)										HT (70% MVIC)									
	First		Early		Mid		Late		Last		First		Early		Mid		Late		Last	
Subject	Slope	Intercept	Slope	Intercept	Slope	Intercept	Slope	Intercept	Slope	Intercept	Slope	Intercept	Slope	Intercept	Slope	Intercept	Slope	Intercept	Slope	Intercept
**1**	−0.05	16.75	−0.05	17.48	−0.04	12.11	−0.05	16.40	−0.04	15.65	−0.06	26.56	−0.04	22.65	−0.03	20.09	−0.04	25.34	−0.03	20.65
**2**	−0.05	18.96	−0.03	16.77	−0.03	17.40	−0.03	16.58	−0.04	17.08	−0.03	16.34	−0.03	17.19	−0.03	18.79	−0.05	22.50	−0.03	19.35
**3**	−0.04	23.34	−0.03	20.25	−0.03	20.14	−0.04	21.94	−0.03	21.16	−0.02	24.90	−0.03	26.07	−0.03	26.91	−0.02	21.81	−0.01	19.70
**4**	−0.04	14.07	−0.02	12.33	−0.02	12.83	−0.04	16.48	−0.03	15.45	−0.03	16.72	−0.02	16.97	−0.04	21.52	−0.04	27.15	−0.04	25.69
**5**	−0.05	23.30	−0.05	23.11	−0.06	23.99	−0.05	23.07	−0.09	27.16	−0.05	20.87	−0.04	18.75	−0.03	18.83	−0.04	21.17	−0.02	19.00
**6**	−0.08	18.46	−0.06	19.55	−0.07	21.03	−0.04	18.80	−0.04	17.85	−0.06	20.79	−0.07	23.30	−0.03	18.19	−0.04	20.56	−0.05	21.84
**7**	−0.02	17.80	−0.02	16.33	−0.01	13.51	−0.02	13.02	−0.02	16.27	−0.01	14.32	−0.03	18.15	−0.02	16.31	−0.03	21.75	−0.02	20.10
**8**	−0.04	15.69	−0.03	15.14	−0.02	14.04	−0.03	14.52	−0.03	16.02	−0.04	16.53	−0.04	16.08	−0.03	16.17	−0.02	14.40	−0.08	26.69
**9**	−0.03	18.03	−0.04	17.68	−0.03	17.07	−0.03	15.71	−0.05	20.18	−0.04	20.79	−0.03	20.65	−0.03	20.33	−0.03	19.67	−0.02	22.06
**10**	−0.03	16.46	−0.03	17.33	−0.03	18.73	−0.03	19.13	−0.03	19.68	−0.03	23.09	−0.03	23.70	−0.04	28.32	−0.03	26.22	−0.03	27.11
**11**	−0.05	22.16	−0.05	20.61	−0.04	18.73	−0.04	18.15	−0.03	17.32	−0.03	22.18	−0.05	25.07	−0.02	23.48	−0.03	26.01	−0.03	25.77
**12**	−0.09	25.70	−0.06	22.43	−0.05	22.10	−0.04	23.43	−0.04	23.91	−0.04	26.31	−0.03	21.54	−0.02	23.54	−0.02	23.07	−0.02	23.86
**13**	−0.03	18.33	−0.06	20.21	−0.03	17.61	−0.03	18.43	−0.05	20.82	−0.03	23.02	−0.04	24.64	−0.05	26.93	−0.04	25.92	−0.03	22.17
**14**	−0.03	17.93	−0.04	18.81	−0.03	16.67	−0.03	18.14	−0.04	17.72	−0.04	18.56	−0.03	20.15	−0.02	19.47	−0.03	22.83	−0.03	24.03
**15**	−0.04	19.61	−0.05	17.91	−0.04	16.84	−0.04	17.13	−0.03	15.42	−0.05	20.09	−0.04	17.67	−0.04	16.68	−0.05	18.50	−0.04	16.96
**16**	−0.05	17.44	−0.04	17.50	−0.04	17.42	−0.04	16.11	−0.05	17.28	−0.06	22.08	−0.06	21.55	−0.04	19.92	−0.09	25.38	−0.03	19.01
**17**	−0.11	22.80	−0.07	19.34	−0.06	17.77	−0.06	19.25	−0.05	18.61	−0.06	24.55	−0.04	20.15	−0.04	18.99	−0.04	22.50	−0.04	20.95
**18**	−0.07	19.11	−0.04	17.10	−0.04	16.42	−0.05	17.07	−0.05	16.42	−0.05	19.00	−0.05	19.74	−0.06	19.78	−0.02	14.13	−0.04	19.53
Mean	−0.05	19.22	−0.04	18.33	−0.04	17.47	−0.04	17.96	−0.04	18.55	−0.04	20.93	−0.04	20.78	−0.03	20.79	−0.04	22.16	−0.03	21.91
SD	0.02	3.07	0.01	2.57	0.01	3.13	0.01	2.75	0.02	3.15	0.01	3.54	0.01	3.00	0.01	3.66	0.02	3.76	0.02	2.99

Inspection of the average MFR versus MUAP_PP_ y‐intercepts (Table 2) suggested a cubic pattern of change during HT exercise (*R* ^2^ = 0.86) characterized by a plateau from the first to early repetition, an increase from the early to late repetition, and a plateau or slight decrease from the late to last repetition (Fig. 4B). There was a quadratic pattern of change for LT exercise (*R* ^2^ = 0.94) that was characterized by a decrease from the first to middle repetition and then an increase from the middle to last repetition (Fig. 4B). The ANOVA analysis indicated that there was no torque × repetition interaction (*F*_4,68_ = 2.26; *P *=* *0.07; $n_{p}^{2}$ = 0.12) or main effect for repetition (*F*_4,68_ = 1.50; *P *=* *0.21; $n_{p}^{2}$ = 0.08), but there was a main effect for torque (*F*_4,68_ = 18.76; *P *≤* *0.001; $n_{p}^{2}$ = 0.53) for the *y*‐intercepts of the individual MFR versus MUAP_PP_ relationships (Fig. 4). The *y*‐intercepts were greater during the HT than LT exercise.

### Motor unit recruitment threshold modulation during fatigue

**Figure 5:**
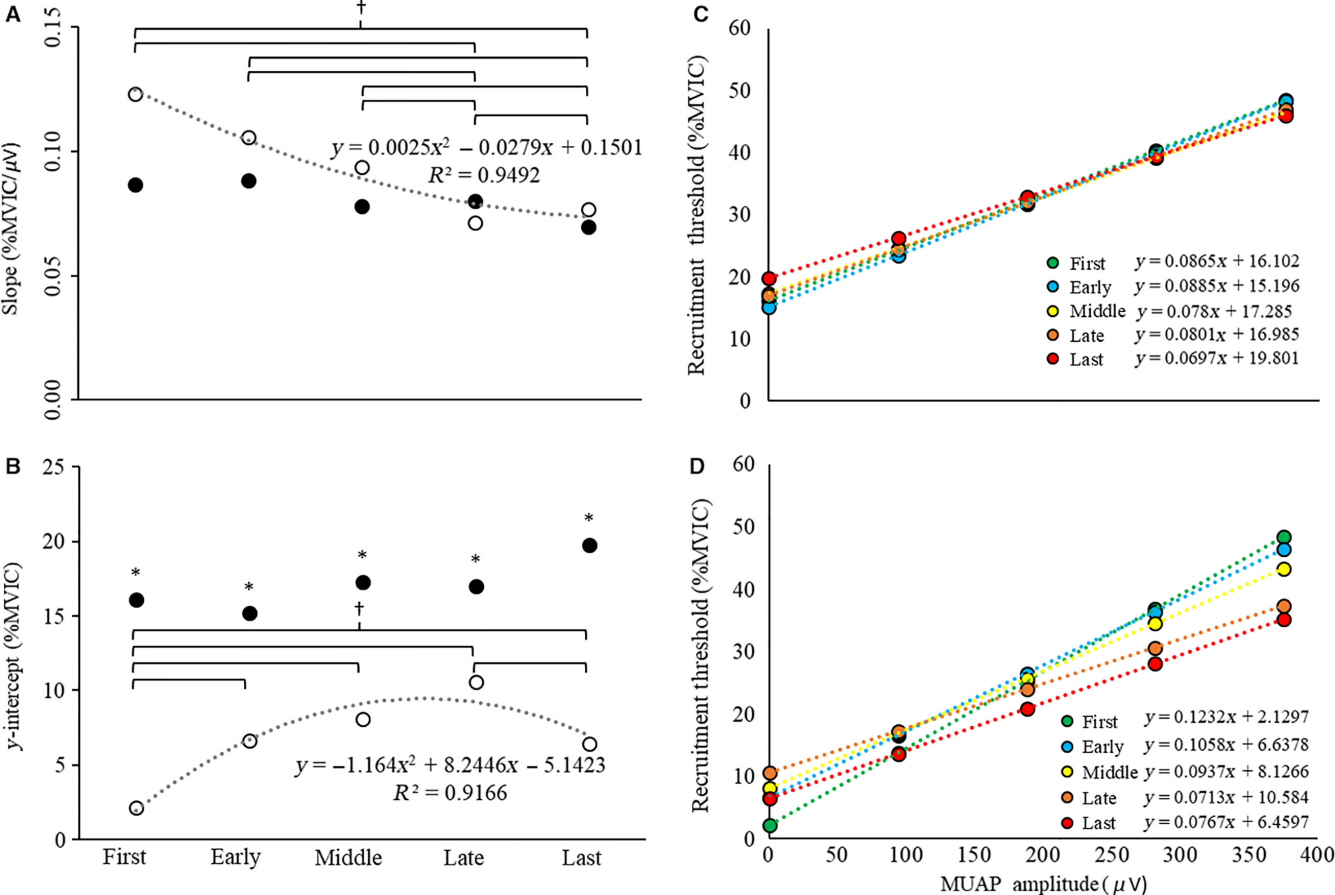
(A–B) The (A) slopes (pps·*μ*V^−1^) and (B) *y*‐intercepts (pps) of the recruitment threshold (RT) versus motor unit action potential amplitude (MUAP_PP_) relationships during the first, early, middle, late, and last repetitions of the high‐torque (solid circles, black dotted line) and low‐torque (open circles, gray dotted line) exercise conditions. (C–D) The average linear regression lines for the RT versus MUAP_PP_ relationship for the first, early, middle, late, and last repetitions during the (C) high‐torque and (D) low‐torque exercise. *indicates a significant main effect for torque (HT > LT); ^†^indicates significant differences between repetitions in the LT condition, only.

Examination of the pattern of change for the average slopes of the RT versus MUAP_PP_ relationships (Table 3) suggested a slight, linear decrease (*r* ^2^ = 0.79) during HT and a quadratic decrease (*R* ^2^ = 0.95) during LT exercise (Fig. 5A). The ANOVA analysis indicated a torque × repetition interaction (*F*_4,68_ = 2.81; *P *=* *0.03; $n_{p}^{2}$ = 0.14). There was no significant change in slope across repetitions during HT exercise (*F*_4,68_ = 1.20; *P *=* *0.32; $n_{p}^{2}$ = 0.07). During LT exercise, the slope did not change from the first to the middle repetition (*P *≥* *0.08), but decreased significantly from the first, early, and middle repetitions to the late and last repetitions (all *P *≤* *0.02; Fig. 5A). There were no significant differences in slope for any repetitions during HT versus LT exercise (all *P *≥* *0.054).

**Table 3:** The individual slopes (pps·*μ*V^−1^) and *y*‐intercepts (pps) of the recruitment threshold (RT) versus motor unit action potential amplitude (MUAP_PP_) relationships during the first, early, middle, late, and last repetitions of the high‐torque (HT) and low‐torque (LT) exercise conditions

	LT (30% MVIC)										HT (70% MVIC)									
	First		Early		Mid		Late		Last		First		Early		Mid		Late		Last	
Subject	Slope	Intercept	Slope	Intercept	Slope	Intercept	Slope	Intercept	Slope	Intercept	Slope	Intercept	Slope	Intercept	Slope	Intercept	Slope	Intercept	Slope	Intercept
**1**	0.17	3.03	0.12	0.48	0.12	10.65	0.14	12.37	0.12	3.95	0.07	23.11	0.07	23.00	0.06	20.70	0.09	12.78	0.06	22.72
**2**	0.09	8.02	0.05	10.50	0.10	10.66	0.09	8.12	0.08	3.94	0.06	16.93	0.08	15.73	0.06	15.51	0.09	14.99	0.10	10.56
**3**	0.07	3.33	0.09	9.20	0.06	7.34	0.07	1.74	0.06	3.83	0.03	21.86	0.04	22.75	0.07	11.39	0.04	22.01	0.03	32.61
**4**	0.08	0.81	0.05	20.36	0.06	15.58	0.08	9.16	0.11	9.88	0.03	20.04	0.03	22.34	0.06	16.11	0.06	15.26	0.07	20.24
**5**	0.06	−1.14	0.18	−7.45	0.21	−8.45	0.12	1.78	0.12	2.44	0.11	8.67	0.11	−0.03	0.09	8.54	0.11	5.31	0.05	18.24
**6**	0.33	0.26	0.20	13.07	0.17	15.70	0.10	15.00	0.11	11.69	0.19	22.98	0.11	32.46	0.10	31.03	0.11	27.04	0.09	38.73
**7**	0.05	5.82	0.06	9.89	0.03	7.27	0.02	9.50	0.03	6.97	0.03	23.57	0.08	15.36	0.07	12.70	0.09	8.92	0.04	14.05
**8**	0.08	15.85	0.06	4.46	0.07	11.27	0.05	13.49	0.08	7.33	0.13	23.69	0.15	17.23	0.13	15.76	0.11	15.73	0.19	−2.67
**9**	0.13	−1.71	0.06	9.03	0.07	7.19	0.04	7.18	0.07	4.68	0.08	9.80	0.11	1.74	0.06	16.05	0.05	23.11	0.04	25.75
**10**	0.09	3.84	0.06	10.90	0.05	11.04	0.04	14.82	0.03	5.93	0.05	16.19	0.07	9.92	0.05	20.88	0.04	22.56	0.04	15.54
**11**	0.16	−5.66	0.15	1.25	0.10	3.00	0.09	4.08	0.04	6.81	0.08	7.08	0.07	5.17	0.04	30.69	0.05	27.36	0.03	30.93
**12**	0.18	−2.96	0.15	2.36	0.07	5.92	0.07	1.48	0.06	2.85	0.10	3.31	0.10	5.12	0.06	9.83	0.03	23.21	0.04	15.48
**13**	0.06	12.17	0.07	12.53	0.04	14.57	0.02	23.03	0.06	3.84	0.09	7.78	0.05	30.25	0.11	12.99	0.08	17.92	0.11	11.58
**14**	0.08	0.27	0.08	10.77	0.06	12.08	0.05	9.65	0.04	7.23	0.06	21.47	0.07	15.79	0.08	9.46	0.04	19.99	0.06	16.02
**15**	0.14	1.17	0.11	13.61	0.11	9.23	0.08	9.24	0.04	8.50	0.11	13.14	0.11	12.13	0.12	15.57	0.18	3.15	0.08	12.52
**16**	0.10	0.55	0.14	−0.66	0.08	15.61	0.03	24.47	0.06	19.22	0.10	14.08	0.10	17.11	0.12	23.50	0.13	14.95	0.06	36.43
**17**	0.23	−4.13	0.13	0.12	0.10	0.77	0.10	17.66	0.16	1.01	0.01	33.37	0.08	26.86	0.08	21.57	0.07	20.19	0.08	27.41
**18**	0.11	−1.18	0.14	−0.92	0.17	−3.16	0.09	7.72	0.11	6.19	0.23	2.76	0.18	0.59	0.07	18.85	0.09	11.23	0.09	10.28
Mean	0.12	2.13	0.11	6.64	0.09	8.13	0.07	10.58	0.08	6.46	0.09	16.10	0.09	15.20	0.08	17.29	0.08	16.99	0.07	19.80
SD	0.07	5.51	0.05	6.98	0.05	6.63	0.03	6.68	0.04	4.18	0.05	8.35	0.04	10.01	0.03	6.56	0.04	6.94	0.04	10.61

Examination of the pattern of change for the average *y*‐intercepts of the RT versus MUAP_PP_ relationships (Table 3) suggested quadratic changes across repetition during both HT (*R* ^2^ = 0.86) and LT (*R* ^2^ = 0.92) exercise (Fig. 5B). During HT, the change was best characterized as a slight decrease from the first to early repetition, followed by an increase from the early to last repetition. Whereas, during LT, the change was characterized by an increase from the first to late repetition, followed by a decrease from the late to last repetition. The ANOVA analysis indicated a torque × repetition interaction (*F*_4,68_ = 2.94; *P *=* *0.03; $n_{p}^{2}$ = 0.15). There was no significant change in *y*‐intercept during HT exercise (*F*_4,68_ = 1.03; *P *=* *0.40; $n_{p}^{2}$ = 0.06). During LT exercise, however, the y‐intercept increased from the first to the early repetition (*P *=* *0.02), plateaued from the early to the late repetition (all *P *≥* *0.08), and then decreased from the late to the last repetition (*P *=* *0.01). The y‐intercepts were greater during HT than LT exercise (all *P *≤* *0.01) for all repetitions.

### Maximal detected motor unit action potential amplitude during exercise

**Figure 6:**
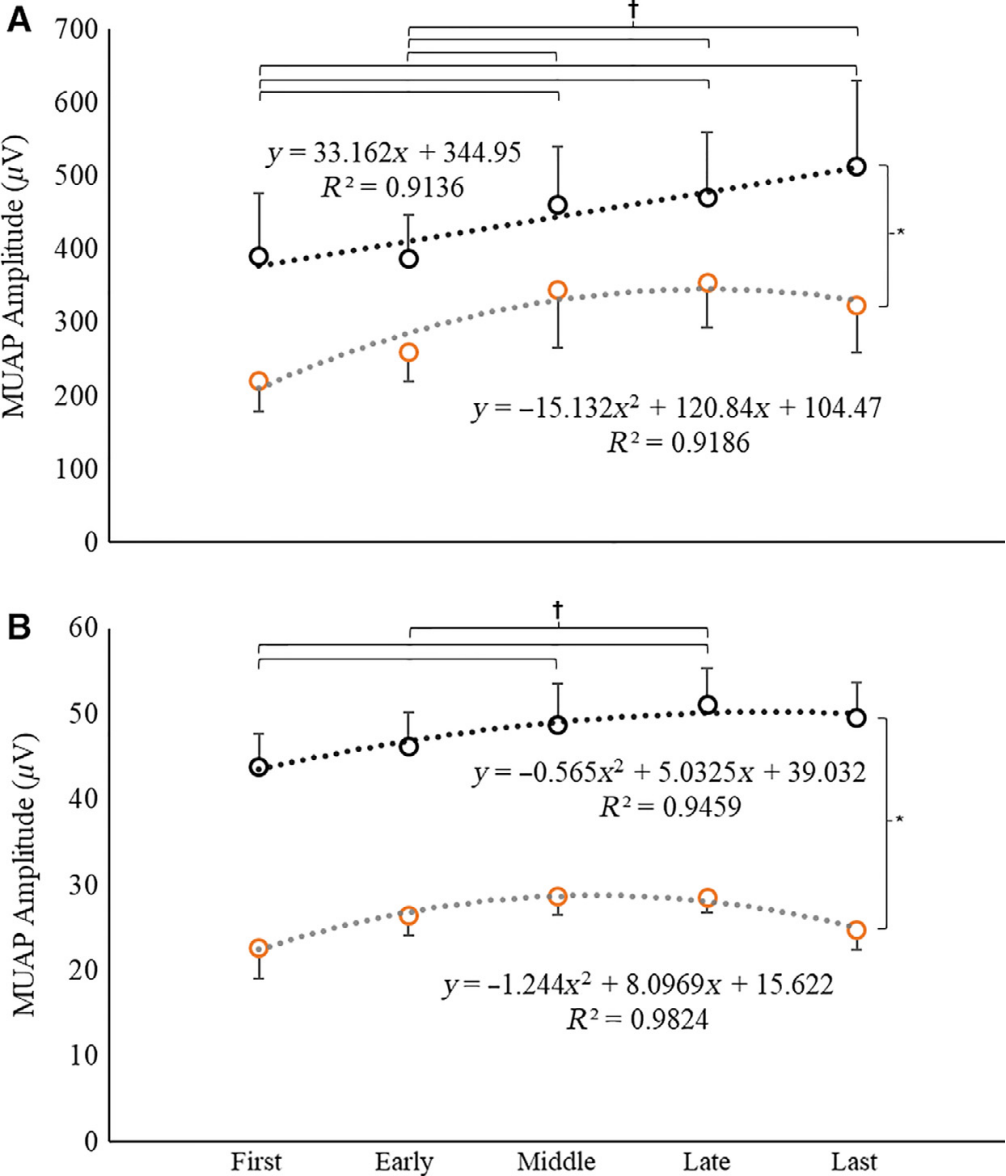
(A–B) The mean (±95% confidence interval) maximal detected MUAP_PP_ and corresponding RT, respectively, in the high‐torque (black open circles, black dotted line) and low‐torque (orange open circles, gray dotted line) exercise conditions. *indicates a significant main effect for torque (HT > LT); ^†^indicates significant differences between repetitions, independent of torque.

**Figure 7:**
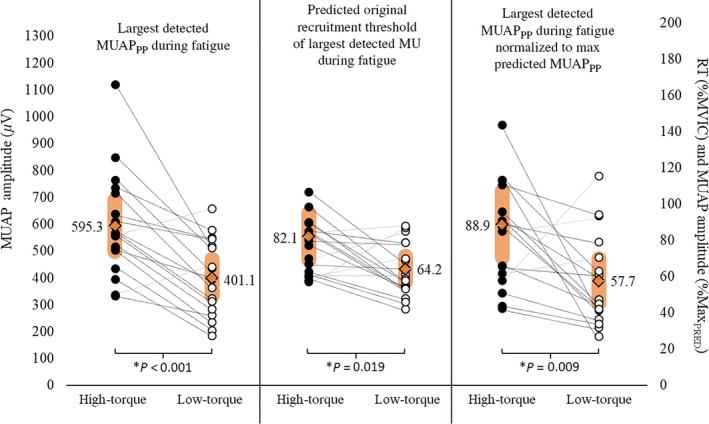
The maximal detected (largest) motor unit action potential amplitude (maxMUAP_PP_;* μ*V) during HT versus LT exercise, the predicted original recruitment threshold (RT; %MVIC) of the maxMUAP_PP_ during HT versus LT exercise, and the maxMUAP_PP_ expressed relative to the predicted amplitude of the maximal (largest) MU (%Max_PRED_) during HT versus LT exercise. The orange diamonds and shaded regions represent the mean ± SD values, respectively, whereas the individual lines connect the data points of individual subjects.

For maximal detected MUAP_PP_ (maxMUAP_PP_) during the first, early, middle, late, and last repetitions (Fig. 6), there was no torque × repetition interaction (*F*_4,68_ = 1.11; *P *=* *0.36; $n_{p}^{2}$ = 0.06), but there were main effects for torque (*F*_1,17_ = 36.02; *P *<* *0.001; $n_{p}^{2}$ = 0.68) and repetition (*F*_4,68_ = 9.16; *P *<* *0.001; $n_{p}^{2}$ = 0.35). Furthermore, maxMUAP_PP_ was greater during HT than LT exercise, independent of repetition (Fig. 7). In addition, maxMUAP_PP_ (collapsed across load) did not change from the first to early repetition, but increased from the first and early repetition to the middle repetition (all *P* ≤ 0.01) and then plateaued from the middle to the last repetition (all *P *≥* *0.56; Fig. 6C). There was no torque × repetition interaction (*F*_4,68_ = 0.91; *P *=* *0.42; $n_{p}^{2}$ = 0.06), but there were main effects for torque (*F*_1,17_ = 377.65; *P *<* *0.001; $n_{p}^{2}$ = 0.96) and repetition (*F*_4,68_ = 4.82; *P *=* *0.002; $n_{p}^{2}$ = 0.22) for the RT associated with the maximal detected MUAP_PP_ (maxMUAP_PP_) during each repetition. The RT associated with the detected maxMUAP_PP_ was greater during HT than LT exercise, independent of repetition (Fig. 6D). Furthermore, RT (collapsed across load) did not change from the first to the early repetition, but increased from the first to the middle repetition and from the early to the late repetition, but was not different from the first repetition during the last repetition (Fig. 6D).

### Predicted recruitment threshold and maximal motor unit size

Figure 7 displays the mean, 95% confidence intervals, and individual data points for maxMUAP_PP_, the predicted original RT of maxMUAP_PP_, and maxMUAP_PP_ relative to each subject's maximal predicted MUAP amplitude. The maxMUAP_PP_ was greater in HT than LT exercise (595.27 ± 95.88 *μ*V vs. 401.08 ± 69.28 *μ*V, respectively; *t*_17_ = 4.95, *P *<* *0.001). The predicted original RT of maxMUAP_PP_ was also greater during HT versus LT exercise (82.13 ± 12.97%MVIC vs. 64.19 ± 7.17%MVIC, respectively; *t*_17_ = 2.59, *P *=* *0.02). Finally, the maxMUAP_PP_ expressed relative to the largest predicted MUAP_PP_ was greater during HT versus LT exercise (88.86 ± 18.39%Max_PRED_ vs. 57.67 ± 12.23%Max_PRED_; *t*_17_ = 2.91, *P *=* *0.009).

## Discussion

To our knowledge, this is the first study to examine and report MU behavior from a large population of active MUs during HT and LT contractions performed to volitional fatigue. There was no difference in MVIC strength prior to exercise, or in the total repetitions and total work completed during exercise between conditions (Fig. 2). However, during LT exercise, participants exhibited a greater time to task failure and, by nature, a more dramatic decline in the muscle's force capacity than observed during the HT condition. During both HT and LT, time to task failure was inversely related to MVIC strength (Fig. 3A and B). We observed the behavior of 4670 MUs across 18 subjects during HT and LT exercise (HT = 1582 MUs; LT = 3088 MUs). The average number of MUs analyzed per contraction was 17.6 (±3.2) in the HT condition and 34.2 (±7.5) in the LT condition. Although firing rates increased throughout both HT and LT exercise (Fig. 4), the MFRs were higher throughout HT exercise than LT exercise (Fig. 4). Furthermore, LT exercise resulted in significant changes in RT across the MU pool, but HT did not (Fig. 5). Regardless, both HT and LT performed to fatigue resulted in the recruitment of additional, larger MUs (Fig. 5C and D) and, despite the more remarkable reduction in RT and corresponding MU recruitment during LT exercise, on average, larger MUs were recruited during the HT than LT exercise.

During repeated submaximal contractions at 30% MVIC performed to fatigue, Contessa et al. (2016) reported a “clear and consistent” increase in MFRs across the MU pool when examining the firing behavior of individual MU firing trains as a function of their MUAP amplitudes. In the current investigation, we utilized a larger sample (*n* = 18 vs. *n* = 5 in Contessa et al. (2016)) and examined MU behavior during submaximal fatiguing contractions at both high‐ (i.e., 70% MVIC) and low‐torque (i.e., 30% MVIC) levels. Similar to Contessa et al. (2016), we observed increases in MU MFRs from the first to middle, late, and last repetitions in both exercise conditions (Fig. 4A). However, as can be seen in Figure 4 and Table 2, the patterns of change for the slopes and y‐intercepts for the MFR versus MUAP_PP_ relationships were different in HT versus LT exercise. Specifically, a uniform increase in MFR was observed across the MU pool during HT exercise, whereas during LT exercise there was no change in MFR for the smallest MUs and an increase in MFR for the largest MUs. Moreover, whereas Contessa et al. (2016) examined changes in MU behavior up to and including a “late” repetition, we also examined MU behavior during the final (e.g., last) successfully performed repetition preceding task failure. During the last repetition, there was a slight decrease in slope of the MFR versus MUAP_PP_ relationship relative to the middle and late repetitions in the LT condition (Fig. 4A). This may have been caused by recruitment of larger MUs. Finally, the firing rates for all detected MUs were higher throughout HT versus LT exercise, which was indicated by greater y‐intercepts during HT exercise, but no difference in slopes (Fig. 4).

In conjunction with the changes in MFR, the slope of the RT versus MUAP_PP_ relationship decreased from the first to late repetitions, before exhibiting an increase from the late to last repetition during LT exercise (Fig. 5A; Table 3). This alteration in slope for the RT versus MUAP_PP_ relationship was accompanied by an increase in y‐intercept from the first to early repetition, a plateau from the early to late repetition, and a subsequent decrease from the late to last repetition during LT exercise (Fig. 5B). These data suggest, therefore, that over the course of LT exercise to fatigue, the RT of the smallest, most active MUs slightly increased and the RT of the larger, newly recruited MUs decreased (Fig. 5D). This divergent behavior of smaller, lower threshold versus larger, higher threshold MUs during submaximal fatiguing muscle actions has been reported in earlier investigations (Enoka et al. 1989; Carpentier et al. 2001; Farina et al. 2009). For example, Farina et al. (2009) reported that the MUs recruited at the beginning of repeated submaximal isometric contractions (i.e., the most active MUs) displayed an increase in RT, whereas MUs that were recruited later in the task and were less active exhibited a decrease in RT. Interestingly, however, there were no significant changes in the slopes and y‐intercepts of the RT versus MUAP_PP_ relationship during HT exercise in the present investigation (Fig. 5A and B). Therefore, as noted by Farina et al. (2009), the relative duration of activity likely results in different adjustments of the MU pool during intermittent, isometric contractions. It is unlikely that this divergent behavior is a result of individual‐changes via central drive to the MU pool, but rather due to motor neuron adaptation, afferent feedback from fatiguing muscle, disfacilatation of the MU pool, or some combination thereof.

**Figure 8:**
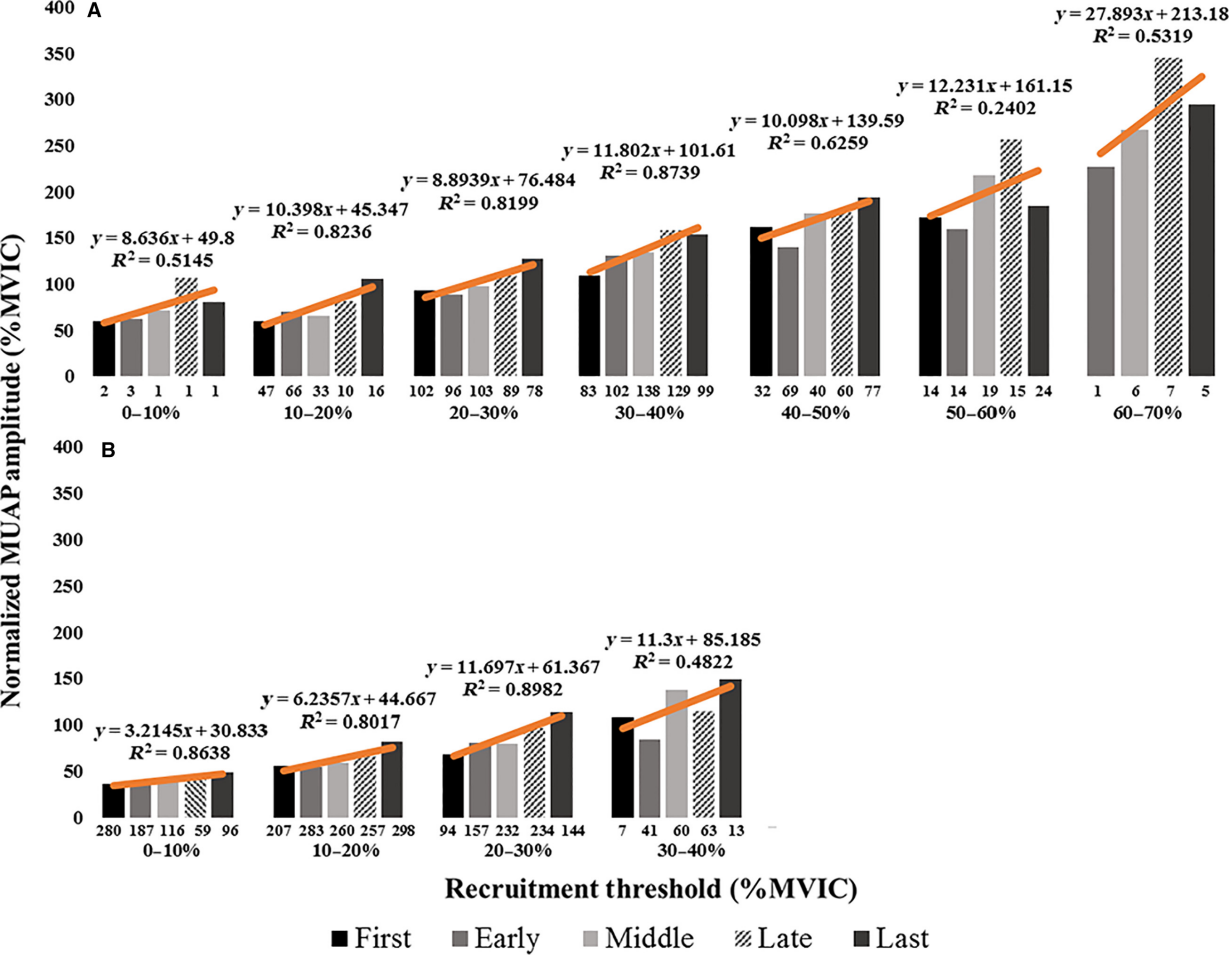
(A–B) The average, normalized motor unit action potential amplitude (MUAP_PP_) during the first, early, middle, late, and last repetitions for motor units recruited within the 0–10%, 10–20%, 20–30%, 30–40%, etc. MVIC force range during the (A) high‐torque and (B) low‐torque exercise conditions. The numbers below each bar indicate the number of motor units analyzed in the corresponding recruitment threshold bin for that repetition. The relationship between average MUAP_PP_ and repetition was fit with linear regression lines (orange lines).

In humans, an increase in net excitatory synaptic input to the MU pool results in the progressive and orderly recruitment of larger, higher threshold MUs (Henneman 1957; De Luca and Erim 1994; Hu et al. 2013a). It is thought that, during fatiguing submaximal contractions, the central nervous system increases excitatory drive to the MU pool in order to maintain force production despite decreases in MU twitch forces (Contessa et al. 2016). Indeed, the results of this study suggest that there was an increase in excitation to the MU pool, as evidenced by the orderly recruitment of larger, higher threshold MUs during both HT and LT exercise to failure (Fig. 8). It has been suggested that full recruitment of the MU pool will occur independent of the force or load used, provided exercise is sustained to volitional fatigue because excitatory input to the MU pool will increase indefinitely to meet the force demand (Mitchell et al. 2012; Potvin and Fuglevand 2017b). Building on this premise, Potvin and Fuglevand (2017a) recently developed a model which predicted that the entire MU pool would be recruited during fatiguing contractions at 20% MVIC. While this model is valid in theory, our results suggest that larger, higher threshold MUs were consistently recruited during the HT contractions (Fig. 6), which was further evidenced by the greater observed maxMUAP_PP_, predicted original RT of maxMUAP_PP_, and maxMUAP_PP_ relative to each subject's maximal predicted MUAP amplitude (%Max_PRED_) during HT versus LT exercise (Fig. 7).

A careful inspection of previous studies which have examined muscle activation during HT versus LT isometric exercise lends support for our findings (Lind and Petrofsky 1979; Petrofsky et al. 1982; Fuglevand et al. 1993). For example, Fuglevand et al. (1993) examined EMG amplitude and maximal compound action potential (M‐wave) amplitude during fatiguing submaximal contractions at 20%, 35%, and 65% MVIC. The authors (Fuglevand et al. 1993) reported increases in EMG amplitude and decreases in M‐wave amplitude throughout each condition that were opposite in magnitude of the sustained torque‐level, although EMG amplitude did not reach maximal values in any condition. These data suggest that it is more likely that maximal muscle activation is obtained if the target force or torque is high than low (Fuglevand et al. 1993). Consequently, Fuglevand et al. (1993) suggested that the “limitation in neural excitation of muscle during fatiguing contractions may be partially due to impaired neuromuscular propagation in addition to factors that reduce the net output of the motor neuron pool” (pg. 563). Thus, it is likely that MU pool output is limited to a greater degree during LT than HT exercise and may explain why larger MUs were recruited in the HT condition in this study.

While larger, higher threshold MUs were recruited, on average, during HT exercise, several subjects recruited MUs during LT exercise that were similar in size and/or original RT when compared to those detected during HT exercise (Fig. 7). Thus, the capacity to recruit the full MU pool during LT exercise to failure may be subject‐dependent. The ability to supply sufficient excitation to the MU pool in order to maintain whole muscle force production during fatiguing, submaximal contractions is probably dependent on competing processes, several of which likely include: (1) increased excitatory drive to the MU pool (Contessa et al. 2016), (2) intrinsic changes in motor neuron excitability (Kernell and Monster 1982), (3) neuromuscular propagation failure (Fuglevand et al. 1993), (4) reflex disfacilitation from a decline in group Ia excitatory input from muscle spindle afferents (Macefield et al. 1991), and (5) reflex inhibition from group III and IV muscle afferents (Bigland‐Ritchie et al. 1986; Woods et al. 1987; Garland and McComas 1990; Duchateau and Hainaut 1993). It is probable that, due to the longer time to task failure for the LT versus HT exercise in this study, each of the factors which may limit MU pool output, such as neuromuscular propagation failure, reflex disfacilitation or inhibition, and decreased intrinsic excitability, have a greater influence on LT than HT exercise to failure. For example, Fuglevand et al. (1993) suggested that there was greater neuromuscular propagation failure during exercise at 20% versus 65% MVIC to volitional fatigue. Macefield et al. (1991) reported that, as muscle fatigues, Ia excitatory input to the MU pool decreases and results in a decline in MFR that is most apparent for MUs with the highest firing rates (i.e., low‐threshold MUs). This attenuation of reflex support to the MU pool is a slowly developing process (Bongiovanni et al. 1990) and may therefore be more dramatic in LT conditions characterized by longer times to task failure. It has also been suggested that during exercise, the central nervous system (CNS) constrains the output of spinal motor neurons, and ultimately muscle activation, to inhibit the development of peripheral fatigue (Amann et al. 2006). Group III and IV muscle afferents, which are sensitive to mechanical and metabolic stimuli associated with muscle contractions (Mense 1977; Kniffki et al. 1978; Rotto and Kaufman 1988; Light et al. 2008) have been reported as a neural link between the CNS‐mediated decrease in motor neuronal output and the degree of peripheral fatigue (Amann et al. 2011; Sidhu et al. 2014). Consequently, a growing body of evidence supports the concept that the end of exhaustive exercise may coincide with an individual‐ and task‐dependent degree of locomotor muscle fatigue (Amann et al. 2006; Romer et al. 2007; Amann and Dempsey 2008; Gagnon et al. 2009; Burnley et al. 2012; Rossman et al. 2014; Hureau et al. 2016) related to the intramuscular metabolic environment (Hogan et al. 1999; Burnley et al. 2010; Vanhatalo et al. 2010; Chidnok et al. 2013) which may have undergone more dramatic alterations in the longer duration, LT condition. Thus, any of these mechanisms, or a combination thereof, may explain why, compared to HT exercise, the recruitment of additional, larger, higher threshold MUs seemed to be constrained during LT exercise (despite some subjects appearing capable of recruiting all available MUs (i.e., greater buffering capacity, lower sensitivity, etc.)), and may also explain the slight reversal in the MU behavioral adaptation seen from the beginning (i.e., first through late repetition) to the last repetition of LT exercise (Figs. 4A and B, 5A and B, 6B and C).

In summary, we report that HT and LT isometric contractions performed to failure resulted in the same amount of work completed, but a longer time to task failure and a more dramatic decrease in the muscle's force capacity was observed during LT exercise. A uniform increase in MFR was observed across the MU pool during HT exercise, whereas during LT exercise the change in MFR was most dramatic for the largest MUs. Furthermore, MFR was higher for all MUs across all repetitions throughout HT than LT exercise. We also observed no significant changes in RT during the HT condition, but over the course of repeated LT exercise to failure, the RT of the smallest MUs increased and the RT for the moderate to large MUs decreased. Both HT and LT isometric contractions performed to failure resulted in the recruitment of additional higher threshold MUs in order to maintain torque production. However, throughout exercise, the HT condition required the recruitment of larger MUs than did LT exercise. Therefore, although low‐torque contractions performed to failure cause recruitment of higher threshold MUs, our results suggest that they do not necessitate the recruitment of the largest available MUs as do high‐torque contractions. In a few cases, however, MUs were recruited by individuals during LT exercise that were similar in size and original RT to those detected during HT exercise. Thus, the ability to achieve full MU recruitment during LT exercise may be dependent on the subject, but further work is needed to confirm these preliminary findings. Consequently, our data further emphasize the task‐ and subject‐dependency of muscle fatigue.

## Conflict of Interest

None declared.
